# Addressing the Challenges of Conducting Observational Studies in Sheep Abattoirs

**DOI:** 10.3390/ani7110082

**Published:** 2017-11-01

**Authors:** Elyssa Payne, Melissa Starling, Paul McGreevy

**Affiliations:** Sydney School of Veterinary Science (B19), University of Sydney, Camperdown, NSW 2006, Australia; melissa.starling@sydney.edu.au (M.S.); paul.mcgreevy@sydney.edu.au (P.M.)

**Keywords:** herding dogs, sheep, livestock, abattoir, welfare, in situ observation

## Abstract

**Simple Summary:**

Collecting data, particularly data on animal behavior, on-site at abattoirs, can be hindered by a series of challenges. These challenges are summarized and recommendations are offered for those planning similar studies. Particular emphasis is placed on examining interactions between dogs, handlers and sheep in animal-processing facilities because this is a significantly under-researched area in the literature. There is significant merit in collecting data via video-recording software, but the subsequent potential for hardware issues and sampling difficulties must be recognized and addressed.

**Abstract:**

The competing needs of maintaining productivity within abattoirs, and maintaining high standards of animal welfare, provide fertile grounds for applied research in animal behavior. However, there are challenges involved in capturing useful behavioral data from the supply chain (from paddock to processing plant). The challenges identified in this report are based on a review of the scientific literature as well as field study observations. This article describes those challenges as they relate to collecting behavioral data on livestock-herding dogs, humans and livestock as they interact in abattoirs, and provides insights and recommendations for others embarking on animal studies in confined spaces, as well as in commercial settings. Direct observation of livestock behavior permits animal-welfare assessments and evaluations of the efficacy of operations in unfamiliar and high-pressure contexts, such as abattoirs. This brief report summarizes the factors that must be considered when undertaking in situ studies in abattoirs. There is merit in passive behavioral data-collection using video-recording equipment. However, the potential for hardware issues and sampling difficulties must be anticipated and addressed. Future research directions and recommendations to avoid such issues are discussed. This information will be highly beneficial to future abattoir studies focusing on efficiency and animal welfare at commercial abattoirs. Furthermore, it may also be relevant to any analyses involving large cohorts of animals in a confined environment.

## 1. Introduction

Abattoirs provide a good example of the challenges involved in balancing both animal-welfare goals and production goals. In Australia, approximately 30 million sheep (including lambs, wethers and ewes) were processed in 2016 [1]. Consequently, many abattoirs process thousands of animals per day and, for commercial and operational reasons, it is important that livestock move through the processing chain at a steady rate. However, the welfare of the animals being moved through the abattoir is also critically important. Not only do we have a moral responsibility to ensure that animals slaughtered to provide us with meat and various by-products are spared unnecessary suffering and distress [2], but low-stress treatment also benefits carcass quality [3].

### 1.1. Why Study Animals in Abattoirs?

In situ studies of animal-processing plants are invaluable because of their authenticity. Compared with laboratory-based studies, they have the advantage of recruiting currently employed stockmen and dogs, thereby ensuring that data collected are applicable to functioning establishments. They can also assess the direct consequences of slaughter procedures and concurrent stimuli (including handler behavior) on stock. There are three significant topical areas that can be investigated on site in abattoirs that would not be possible to simulate in a laboratory. First and foremost, relatively under-researched interactions, such as dog-livestock interactions in abattoirs, can be analyzed and their advantages and disadvantages reviewed. The use of dogs in abattoirs is particularly pertinent given herding has been significantly associated with distress in sheep [4]. Second, the balance between animal welfare and efficiency of operations can be assessed in any given establishment. Third, behaviors indicative of compromised livestock welfare can be identified and subsequently used to monitor animal-processing facilities.

### 1.2. Animal Welfare and Productivity

Balancing animal welfare and productivity in commercial abattoirs provides significant challenges that can be met, in part at least, with the assistance of applied science. Optimal handling of livestock in environments that are often both alien to the stock and somewhat confining to both livestock and those moving them requires the assessment of both animal welfare and the efficiency of operations. For such assessments to be valid, several factors must be considered, including the efficacy of livestock-handling strategies, the stimuli that trigger distress and influence rate of movement, and livestock behaviors that indicate distress [5]. Accordingly, in the light of these potentially competing objectives, the optimal approach ensures that livestock move when required at a rate compatible with productivity goals, while also protecting the animals from needless distress. Although, at least conceptually, addressing both these aims satisfactorily may seem straightforward, abattoir design may contribute positively or negatively. Abattoirs are not necessarily designed by people who understand the principles of livestock movement, and designs may be constrained by available funds, available space, and existing structures. Indeed, abattoir design and the human-animal interactions that occur within abattoirs can be determined by legislation and culture, rather than by empirical studies of ethology and animal welfare. In some cases, variations in design may introduce areas that livestock are reluctant to pass, or long races in which livestock may lose momentum or that increase the workload of stock-handlers per shift [6]. In such cases, livestock-herding dogs may be used to assist in livestock movement in the abattoir.

### 1.3. Knowledge Gap: Dog-Livestock Interactions

In some countries, such as Australia, dogs can be used to assist with livestock movement in lairage. Recent studies of livestock-herding dogs in Australia have revealed both the value of these dogs [7] and the shortage of empirical evidence to characterize best practise [8,9,10,11]. The welfare and productivity of the dogs have been the focus of these studies, while the welfare of the stock they herd may have been peripheral. Nevertheless, the triadic nature of humans and dogs herding livestock has profound implications for production-animal welfare. Optimizing stockmanship, dogmanship and the interactions between permits the evaluation of the overall use of dogs in abattoirs. This is particularly pertinent given that the use of dogs has been identified as a significant stressor of sheep [12]. Consumers of animal products expect that high standards of animal welfare are established and maintained for livestock [2]. Additionally, there is increasing evidence of demand for ethically produced food [13,14]. So, assessment of animal welfare both on farms and in abattoirs is needed to ensure that high animal-welfare standards are maintained at all stages of livestock production, which is vital for keeping the industry sustainable. Interactions between dogs and livestock represent a gap in current knowledge of livestock welfare, particularly in abattoirs where, arguably, this can be more readily assessed in a systematic way than on farms.

### 1.4. Behavioral Welfare Indicators

Behaviors indicative of poor welfare or sub-optimal handling practises should be included in regular animal-welfare audits [15], which could also inform training and extension programs to promote humane stockmanship and have been shown to improve on-site animal welfare [16]. Of course, while these audits may succeed in maintaining a minimum welfare standard, they may not reveal optimal welfare practises. As such, comprehensive animal welfare studies on site in abattoirs are needed. Observations of operations in lairage (on-site facilities that house animals prior to slaughter) can illustrate typical human-animal and dog-livestock interactions that stock are exposed to as well as how stock are moved from lairage to the point of slaughter (the so-called stunning box). These observations can also help to reveal the efficiency of livestock handling and processing facilities. Outcomes can include behavioral variables relevant to efficient livestock movement, such as bunching and stalling, but also carcass attributes and concentrations of hormones in blood collected immediately after death [12]. Such research quantifies direct consequences of certain stimuli or events, so that any policy or management changes introduced in light of the resultant evidence may be more readily embraced. Given that product quality, profitability and welfare are linked [17,18], objective assessment of operations in abattoirs and lairage is essential if facilities are to operate efficiently and prioritize animal welfare.

This article explores the benefits and limitations inherent in applied animal-behavior studies in commercial abattoirs based on the current literature and observations from recent field studies (unpublished data). In many countries, the number of human-livestock, dog-livestock and human–dog interactions that occur in such establishments is considerable. To provide specific examples, the article has a focus on sheep abattoirs using dogs to move stock. It does not dwell on the ethical considerations of monitoring the behavior of abattoir workers. Information contained in this article can be of use to individuals wishing to observe interspecific interactions in abattoirs and assess the efficiency of stock movement.

## 2. Materials and Methods

**Figure 1:**
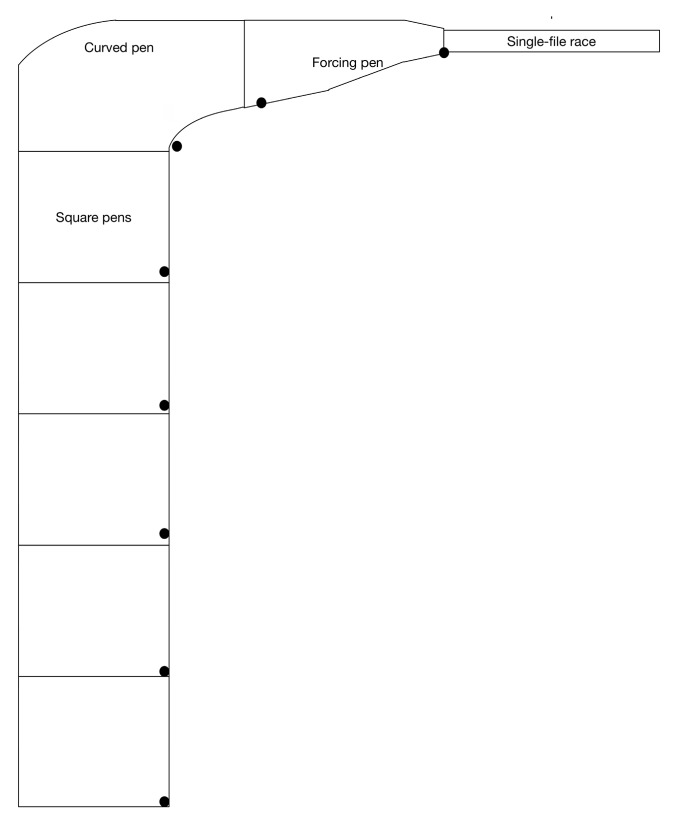
Diagram of the area observed at an Australian abattoir. Black circles indicate camera locations. Cameras were arranged in order from Camera 1 in the square pen at the bottom of the diagram through to Camera 6 at the curved pen, Camera 7 at the forcing pen, and Camera 8 at the single-file race.

This report was informed by both a review of the literature and observations gained from recent data collection studies at an Australian sheep abattoir. Sheep, handler and dog behavior was recorded over a three-day period as animals were moved through a series of square yards used for lairage, followed by a curved race that funnelled the sheep into the forcing pen and, lastly, into the single file race. A diagram summarizing the abattoir layout and camera placement is provided in Figure 1.

### 2.1. Subjects

Over 3000 sheep and a series of stock handlers and their dogs were observed in an Australian sheep abattoir.

### 2.2. Behavioral Observations

Ethograms for handlers, dogs and sheep were developed after consulting those published in previous relevant studies [12] and appear as Appendix A Table A1, Table A2 and Table A3. Behaviors of interest were those that were directly related to welfare (sheep ethogram) or influenced sheep behavior and movement (dog and human ethograms).

## 3. Challenges in Abattoir Studies

Despite the many benefits offered by in situ abattoir studies, several factors, according to the literature and recent field studies, have the potential to impede data collection and analyses. For results to influence large-scale policy or change management practises, studies need to be valid, repeatable, reliable and feasible [19]. Subjects and abattoir designs can vary markedly, making it difficult to generalize with the findings. Additionally, researchers must also take care to avoid observer effects and immediately resolve any technical issues that could arise if they are not on site. The sampling level, the method of collecting behavioral data and subsequent data of interest should also be considered and balanced.

### 3.1. Subject Availability

Studies in commercial abattoirs have the disadvantage of the stock subjects being terminated, so any cumulative effect of different treatments or stimuli on individual animals cannot be examined in the mid- to long-term. Furthermore, given the dynamic nature of commercial abattoirs, stimuli cannot necessarily be presented in a standardized fashion at all abattoirs [12], complicating comparisons between different processing treatments or techniques. In cattle, behavioral responses to stress or aversive events (such as vocalisations) have been shown to vary with sex and reproductive status, but not breed [20]. Sheep stress responses (such as vigilance behaviors) in lairage can vary with the animals’ age, breed, source and handling histories [21,22]. To obtain the most meaningful results, studies may need to obtain information on the source and histories of ovine subjects. Furthermore, the skills of individual handlers and individual dogs (when used) may vary greatly [23] and identifying and quantifying such skills is likely to be very challenging without controlled ex situ studies comparing differences between handler-dog combinations.

### 3.2. Abattoir Design and Infrastructure

In commercial abattoirs, animals are typically moved through several adjacent yards that can be separated or combined by opening and closing gates. From the yards, animals are moved into a forcing pen (a curved yard) that funnels them into a single-file race [12]. This single-file race enables animals to be immobilized or stunned individually in the stunning box. Ideally, observational data should be acquired from all sections of an abattoir (from unloading bay to stunning box) to comprehensively capture all human-animal (and dog-livestock, where applicable) interactions that may occur. However, it must be taken into account that there can be considerable variation in the design of abattoirs [24]. Analyses of animal-processing operations should also consider the differences between small- and large-scale plants, with the latter often being adapted to suit processing of multiple species. Consequently, observations of livestock flow and interspecific interactions at one abattoir may not be applicable to others. It is therefore recommended that metrics of stock movement are independent of distance traversed, number of sheep in each pen, or abattoir configuration, as all these factors may vary between abattoirs.

Likewise, speed of movement may be a poor metric of plant efficiency because it may predominantly reflect staff numbers and equipment design [14]. Preferred metrics are those that generalize to other abattoirs, as yet unspecified, because they could overcome differences in size, species processed, equipment and staffing. For example, rate of movement on a per-minute basis is not dependent on distance, but may be influenced by abattoir design and would need to be tested in a range of abattoirs before it could be considered a good metric. The frequency of particular behaviors indicative of distress is not dependent on staff numbers or abattoir design, but may flag poor design when these behaviors occur at high rates. The way sheep and cattle use the space available (e.g., how bunched they are) is considered to reflect anti-predator behavior [25,26] and may serve as a proxy for both distress-related behaviors and rate of movement. Thus, focusing analyses on the frequency of distress-related behaviors offers the potential to provide insight into abattoir efficiency and animal welfare, while accounting for variation among abattoirs. Insight gained from such studies, in conjunction with data on standards of practise, may reveal areas in abattoir design in need of improvement. For example, if animals consistently stall in one particular area along a race regardless of environmental, canine or human factors, it is likely that the race’s design in that area is problematic. Researchers should also note the interplay between dog, human and sheep behavior and consider the prospect that especially intense aversive interactions (such as direct contact between dog and sheep) could be avoided by improved design.

### 3.3. Observer Effects

Advances in video technology and the increasing affordability of cameras has opened the door for data collection when researchers are not on site. Security cameras can produce high-quality footage, be centrally operated, and positioned to ensure that all activity in the pens and single-file race are visible. Recording equipment can be configured to record in the absence of researchers, which allows passive collection of behavioral data. The advantages of researchers being absent at the time of processing are two-fold. First, animals may balk and otherwise react aversively to any unusual presence or movement. As such, any observer’s presence could compromise stock movement, subsequently leading to artefactual changes in stockperson and dog behavior. Second, the presence of observers could potentially lead to a Hawthorne effect, where stockpersons becoming aware of being watched modify their behavior [26]. As video-recording subjects has been shown to counteract the Hawthorne effect [27], in any bid to capture typical stockperson behavior, passive data collection is preferable. The disadvantage of this approach is the risk of hardware difficulties going unnoticed and so unrectified during recording, thus rendering some video files unusable. However, it is important to note that, given the historic use of “leaked” abattoir footage in the media, and the potential deleterious commercial consequences that may ensue, the Hawthorne effect may persist in contexts deemed to be controversial. By the same token, under Australian animal ethics committee approvals, any evidence of criminal activity (including animal abuse) that is revealed as an inadvertent result of research must be reported to the authorities.

### 3.4. Video-Recording Issues

Some technical difficulties may arise with the use of multiple cameras. For example, cameras may develop technical errors, such as image-clarity issues from random filter additions (such as a coloured filter). Additionally, some hardware may be prone to spontaneous restarts, generally after interruptions to mains power supply. Surveillance cameras need to be linked to a central hard drive and operating system, so the sensitive electronic equipment must be powered and housed close to the cameras. This may present a problem in some abattoirs where it may be difficult to find a suitable location for equipment such that it is protected from the elements, extremes in ambient temperature, and interference from physical jolting or fluctuations in the power supply. Likewise, most cameras need to be connected to the central hard drive via a CAT5 cable. This can limit the distance at which cameras can be placed from where the hard drive is kept while on site and can demand dedication of considerable time at setup to ensure that cables will be protected and will not present tripping hazards or otherwise interfere with the movement of workers.

Analyzing affected data after a technical obstruction and tracking focal sheep in subsequent video files can result in a significant burden on observer time, as the focal group is tracked-down. Individual cameras can go offline for short periods while video data are packaged and a new file is prepared. This, in addition to an irregular power supply, can also result in missed transitions between cameras, loss of behavioral data, and delays in later coding associated with tracking the focal group. Depending on the sources of stock arriving at the abattoir, groups of stock in adjacent pens can be very similar in number and appearance, adding to the challenge of locating a focal group if a camera goes offline at a critical time. Furthermore, video data files may be corrupted, resulting in further losses of data. Pre-screening all videos associated with randomly selected focal groups simply to ensure all videos are present and uncorrupted is time-consuming and may not be justified if the risk of missing data is low. In this case, losing track of a particular group of animals may be difficult to avoid, as corrupt files are not always apparent until attempts are made to open them. Taken together, these issues lead us to recommend that, if collecting data with no researchers on site, video-recording equipment must be at the very least tested prior to data collection. Consideration should also be given to the state of the power supply that will be available on site, and measures taken to protect all equipment from the elements and physical interference. Ideally, researchers should have a remote connection to video recording equipment, thereby permitting immediate action in case of technical errors.

### 3.5. Behavioral Data Collection

Cameras can be positioned to capture activities that occur between holding yards and the stunning box. Although researchers may be briefed in advance on the subjects that should be present during recordings, the dynamic nature of animal-processing facilities can still lead to unpredictable events such as unexpected subjects. Fortunately, the appearance on the video record of additional, unexpected dogs and humans can be simply added to the behavioral coding software. However, due to the previously mentioned camera issues, it may be hard to differentiate some subjects from others, especially if dogs are switched at any time. Future studies should gather relevant information through the use of a survey given to stockpersons at the end of each shift, asking for details of dogs used, their breed and the occurrence of any unanticipated events (such as sheep being injured) during their shift.

High camera positions overlooking pens are important for capturing as many animals in a focal group as possible, and therefore, as much behavior in the focal group as possible. However, this may not favor the detection of all behavior. For example, the gait often labelled “stalking” may be defined by low body carriage [9], which is difficult to judge from a high camera angle. Dog movement is often too fast to follow at normal video speed. Slowing video playback gives researchers more time to identify and code behaviors, but does not change the video’s frame rate. Dogs sometimes move so suddenly that the putative cue(s) for them to move are lost when recording at standard frame rate (e.g., 24 frames per second (fps)). Furthermore, slow playback can obfuscate the gait the dog has adopted, particularly if the dog is on screen for only a few seconds. Where possible, attempts to record the behavior of animals that may move quickly for video-coding purposes should be at high frame rates (e.g., 60+ fps).

It may be valuable to collect information on frequency of behaviors believed to be indicative of distress both at times when distress is anticipated and at times when the animals are at rest. A good behavioral indicator of affective state may arise when the affective state is present, but should be absent when the affective state of interest is absent [28]. This approach serves to highlight the value of behavioral data collected when animals are stationary and have had no interactions with dogs or personnel for several minutes.

### 3.6. Sampling

Commercial abattoirs usually process an enormous number of animals. This abundance of data becoming available over a very short time-frame requires the adoption of a sampling method that reduces the amount of data to code and analyze, but ensures that data are obtained evenly across the data-collection period and are representative of the facility’s operation in general. To ensure scoring is blinded, it is also important to remove any potentially confounding data labels prior to coding. As already discussed, the origin and breed of livestock may affect how they behave in an abattoir. Likewise, other variables such as the time of day they are moved, the staff and dogs working the shift, and the ambient conditions may also influence behavior [23,29], so sampling across all these variables is recommended to obtain a comprehensive representation of livestock, human and dog behaviors as they occur in abattoirs.

Animals may be sampled at random as focal animals [24], or as a focal group, with the occurrence of any behaviors from any group member being recorded [12]. If surveillance-type video data are collected, focal cohorts may be determined via start times from a number generator to nominate start times for observations of a set duration. While random sampling is important to remove selection bias in behavioral analysis, it can introduce unanticipated variations into the pool of behavioral data. For example, sampling may include animals that are moved through all pens under surveillance in a single shift as well as those moved only part of the way through the pens under surveillance, before being left overnight and moved through the remaining pens the following day. Excluding the latter groups from sampling would restrict the sampling times so that livestock moved later in the shift would never be sampled. However, any decision to sample animals that are processed over more than one day may introduce the potentially confounding factor of their spending time overnight in one of the pens close to the forcing pen. The livestock included in abattoir studies can have variable handling histories, so some variation between cohorts of animals is to be expected. Accordingly, researchers can expect some inter-cohort variation in behavioral reactions to stimuli.

Historically, abattoir studies have often adopted the approach of recording one or several focal animals rather than a particular focal group [24,30]. However, there are several clear advantages to studying focal groups rather than focal animals. A focal animal may react to actions (e.g., from a dog) directed towards another animal. Recording behavior of a group captures how responses and their frequencies affect the behavior of animals nearby. This may be an appropriate way to examine behavior in a herding species. Furthermore, examining whole groups has the advantage of capturing all potential evidence of compromised welfare and efficiency, thus avoiding the risk of distressed animals going undetected because they were not the focal animals. It may be that some individuals respond differently to stressors than others [29], and this may affect the dataset if particularly reactive individuals are responsible for most of the distress-related behavior recorded.

Following focal groups from one pen (and therefore one camera) to the next can provide challenges related to both technology and the abattoir’s specific livestock. For example, all cameras may go offline simultaneously for a short period while the system reboots following an interruption to the power supply. Video records that have gaps of just three minutes can result in animals being “lost” as they are moved to other pens during the reboot. Clearly, the longer the reboot, the further the animals of interest could have been moved through the processing plant. Furthermore, focal groups generally remain intact for the first few pens, but are often combined with other livestock in pens closer to the stunning box. This can make it difficult to follow a focal group, and therefore difficult to rely on information provided by the abattoir regarding the putative source of the animals. Abattoir workers may also mark the last two or three individuals from a given origin to signify the end of a cohort of a certain breed or source, but it can be difficult to match events such as marking with the information on paper provided by the abattoir.

The movement of focal animals or groups can be followed through all yards before the forcing pen and then through the forcing pen and the single-file race to the stunning box. Following the entire journey of animals from lairage to stunning box captures all human-initiated and dog-initiated behaviors that sheep may show before slaughter. Thus, a comprehensive measure of movement, efficiency and welfare is enabled. However, this approach is likely to increase the need for cameras and cable, so its merits should be carefully considered against the feasibility of recording the entire journey and the value of the extra data it would collect. Recording the entire journey could still fail to capture interactions of interest that may occur when sheep are unloaded from transport. Stunning and slaughter of animals need not be observed if the focus is on livestock behavior, as the animals are often tightly restrained at this point. It is possible to prioritize areas of the abattoir to obtain the most data with the minimum equipment requirements and coding time. Predictably, bottlenecks in movement occur at the single-file race and forcing-pen as animals are funnelled from a group into single-file. Other bottlenecks may occur according to the peculiarities of a given abattoir’s design. To keep them moving, livestock may be kept under near-constant pressure at bottlenecks. Thus, behavior at rest or while not under immediate pressure from humans or dogs may not be considered of less relevance if the current research questions are focused solely at these locations. It is recommended that studies focus on risk factors for such bottlenecks, but also include a control location (pen) where livestock are left stationary while slower parts of the abattoir are cleared. Consideration should also be given to the particular driving methods used by stockpersons at each abattoir as well as the frequency of their application (such as the use of a bell or other noise-making device) at different positions along the race.

### 3.7. Data of Interest

The ethograms used in abattoir studies must be informed by significant behaviors noted in other studies or expert opinion. Care should be taken to observe behaviors that are established indicators of arousal or discomfort, and are hence able to be used in assessments of welfare across different establishments. For sheep, behaviors such as head position [12,29], ear posture changes [31] and freezing [29] are reliable indicators of welfare. Occurrence of these indicators is also likely related to current welfare and not historic welfare on-farm [32]. That said, observing head position and ear posture changes may be difficult in the large and densely packed groups of animals in abattoirs. As such, gross locomotory behaviors, such as freezing or stalling, are more suited to in situ behavioral analyses than behaviors restricted to the head or face.

Similarly, attention should be paid to the visibility of behaviors of other species included in the ethogram, and their relevance to the current research question. For example, observing human gaze direction may be relevant if the effect of this on livestock or dog behavior is of interest, but it may prove difficult to observe if handlers wear accessories such as dark sunglasses or caps, obscuring gaze direction. While human gaze has not been identified as a behavior that influences livestock welfare, dogs do respond to human eye contact as a communicative gesture [33]. The occurrence of this potential cue may elude capture by security cameras set on a steep angle. Additionally, in some areas of the abattoir, dogs and handlers may interact with the same livestock cohort as it spreads across two or more cameras. Any communicative interactions (such as gaze and hand signals) that might span multiple camera fields, such as a signal from one camera directed to a dog currently in view only on another camera, may suffer from missing data. Therefore, there is merit in considering coding data from adjacent videos simultaneously, if that option is available, to capture any dog-human distance communications. A signal or response may be out of view for one camera but in view for another camera, so it may well be picked-up in concurrent observations from neighbouring devices. The use of timestamps facilitates the coding of overlapping observations but care should be taken to avoid the possibility of behavioral events being coded twice if they are observable on two separate video files. Some behavioral coding software programs may enable coding of more than one video file at once, but video files may need to begin with the same timestamp if there is no ability to match timestamps between videos. This may not be possible with security cameras that are designed to automatically start files and are rarely synchronized.

The direction of dog gaze may indicate a dog attending to particular objects, whether human, another dog, or livestock. However, dog gaze directed towards livestock is likely to be unenlightening. If herding dogs are near livestock, it is likely that they are almost always looking at them. Informative behaviors are those that vary and are responsive to changes in the environment and the behavior of other animals or humans, so dog gaze directed towards livestock does not fit these criteria. It may be more informative to record when dogs look at another dog or a handler, because these events occur less frequently and therefore may be more meaningful. They may also be easier to identify, as there are few dogs and humans present and many livestock, and it is less likely that a dog’s gaze in the direction of dogs or humans can be mistaken for another behavior, such as gazing at livestock. More emphasis should be placed on examining state behaviors, such as distance from stock and gait type, as these will be dynamic and vary with a range of behavioral and environmental factors. The intensity of dog use has been linked to compromised welfare of sheep [12]. As such, dog behaviors likely to cause significant distress to sheep (such as stalking, barking and biting) should be monitored [34]. The occurrence of these highly aversive behaviors can then be combined into a composite metric of dog use in abattoirs, thus allowing the evaluation of the use of dogs in these contexts.

Researchers may also consider Qualitative Behavioral Assessments of livestock to obtain a comprehensive indication of animal welfare, beyond the simple absence of any overt distress [35]. Assessing emotions such as brightness, distress and anxiety qualitatively has shown considerable inter-rater reliability [36], and often corresponds to physiological [37] and quantitative behavioral data [38]. As such, Qualitative Behavioral Assessments may be used to highlight positive emotional states in abattoirs, thus optimizing livestock welfare.

## 4. Conclusions

The demand for high welfare standards in the production of animal-based food products, confirms that in situ studies of animal behavior throughout the supply chain (from the paddock to the abattoir) are highly valuable. This paper considers the merits and challenges of in situ behavioral studies in animal-processing plants. The use of video recording software, in particular, offers an opportunity for collecting valuable behavioral data on site, while reducing the chances of operators interfering with stock movement or inadvertently prompting personnel to modify their normal practises. However, rigorous testing and the establishment of backups should accompany all video-recording protocols, to limit missing or unusable data. Comprehensive behavioral observations of livestock groups as they progress through an abattoir, along with the human and dog behaviors they may encounter, is promising. However, in planning such studies, the limitations of collecting data in this environment, including the potential for missing data and the inability to capture some behaviors, must be addressed. Our recommendations should pave the way for future abattoir studies to be conducted efficiently, and optimize the gathering of data relevant to animal welfare and operational efficiency. These recommendations may also be generalized to any analysis of large groups of animals, such as those in intensive production systems.

## Appendix A

**Table A1:** Sheep Ethogram.

Behavior	Description	Rationale for Collecting these Data	Category
Head down	Sheep lowers head so its eyes are below level of the point of the scapula One record for every incidence regardless of individual sheep.	Hemsworth et al. [12] show that lowering the head below this point was significantly positively correlated with elevated cortisol.	Distress
Mount	Both front two legs come off the ground and at least one is placed on top of another sheep. One record for every occurrence on camera.	Probably occurs when sheep are under inescapable pressure.	Distress
Down	Sheep is in either lateral, ventral or dorsal recumbency. One record for every occurrence.	Often occurs in non-resting sheep when they are bunched very tightly. Risk of injury, bruises, stress	Distress
Leap	All four feet are off the ground and the sheep is momentarily airborne. One record for every occurrence on camera.	Escape behavior, associated with startlement or feature of ground surface that the sheep aims to avoid (e.g., high contrast flooring)	Distress
Circling	A total of 80% or more of the sheep in the pen are in physical contact with another sheep. 10% or more of the group is moving in either a clockwise or anti-clockwise direction, with moving sheep consistently turning in towards centre of group. Each moving sheep is in contact with another moving sheep, forming a circle.	Bunching may be comforting to sheep. However, if they start to move away from a stimulus they may fail to get far enough away from it for comfort. In the absence of consummation, the circular movement may become relentless.	Distress
Staring	At least one sheep’s gaze is fixed on a dog or human. Sheep may glance away, but for only an instant before fixating again.	Vigilance towards a particular stimulus probably indicates that stimulus is threatening or interesting.	Distress
Turn back	Sheep at the front of the mob turns around to face the back of the mob and moves against the flow.	This reflects poor facility design because it primarily occurs when sheep see no way forward, or when something in front of them disquiets them [6].	Distress
Jam	In opening to single file race or within the single file race itself, where sheep cannot go forwards and sheep in front cannot go back because two or more individuals are jammed together against the sides of the race.	A jam decelerates the flow, and may cause sheep behind to turn back. Sometimes direct human or canine intervention is required, so sheep are touched or pushed.	Distress
Empty race	Applicable only to single file race, where the last sheep’s hindquarters remain visible at the top of the frame, but no sheep can be seen at the bottom of the frame.	Reveals interrupted flow in the single file race, primarily because of a jam at the forcing pen.	Distress
Head under	One sheep lowered its head and then moved forwards so its head is under the ventrum of another sheep. Indicated by one sheep being lifted off front or hind feet by the sheep underneath.	Similar to head down, but the sheep has also moved forwards while in close proximity to other sheep.	Distress
Foot stamp	Sheep lifts one front foot and brings it down to the ground forcefully in the same place, without moving other feet. One record for each occurrence.	This is considered a defensive behavior, usually in the presence of predators [25].	Distress
Slam	Running sheep makes sufficient impact with infrastructure that they rebound off it.	This may bruise carcasses (most commonly at the flank or on the chest), and is typically seen when the sheep is fleeing from a dog.	Distress
Back up in race	Applicable only to single file race, where at least one sheep in frame reverses with both front and hind feet so that at least one full stride is taken backwards.	Amounts to disrupted flow in single file race, either as a result of a stock person moving ahead of or alongside sheep.	Distress
Kick	Sheep hind foot strikes out behind, with associated leg fully extended, and makes contact with dog, human or another sheep.	Recorded rarely, but presumed to be an offensive behavior coupled with escape.	Distress
Clearing pen	From when gate is opened until either pen is clear of sheep or gate is closed again.	This provides a measure of the latency to clear a pen.	Movement
Stationary	Sheep are waiting in a pen and not being actively moved to the next pen or stage.	This provides a measure of the duration of sheep as they idle in a pen before being moved, and whether this rest period affects how easily they can subsequently be moved.	Movement
Free	Sheep occupation of space is such that there is more than one sheep-body-width of empty space in each direction surrounding at least 80% of the sheep in the group.		Space
Loose	Sheep occupation of space is such that there is approximately one sheep-body-width in two directions surrounding at least 80% of the sheep in the group.		Space
Moderate	Less than one sheep-body-width in at least one direction surrounding 80% of sheep in the group, but sufficient empty space around the sheep to allow sideways or forwards movements that create the space for a single sheep in the group to pass between two other sheep in the group.		Space
Bunched	No visible empty space between sheep flanks, but empty space is visible in front or behind of 80% of the sheep in the group.		Space
Packed	No empty space visible in any direction surrounding at least 80% of the sheep in the group.		Space

Ethogram used for the coding of sheep behavior in an in situ pilot study of an Australian abattoir.

**Table A2:** Dog Ethogram.

Behavior	Description	Rationale	Category
Rush	Dog rushes from outside flight zone for a distance of at least one-sheep-body-length towards sheep and may snap or jump at them.	Dogs that do this are penetrating well into the sheep’s flight zone and sheep may not have the opportunity to escape from them.	Force
Parked	One or more dog is stationary in same pen as sheep. Dog may make adjustments in position involving less than two sheep-body-lengths.	The stationed dog is passive, but puts pressure on sheep by its presence. If the dog is stationed too close, the sheep may mount or turn to face the dog.	Pressure
Back	Dog jumps onto and travels over backs of sheep.		Pressure
Walking	Dog is walking, defined as a 4-beat gait.		Dog movement
Stalking	Dog is stalking, with the body lowered, head up or lowered and extended forward, ears erect, on top of head, and pointing forward, tail below back level and motionless. Forward motion is slow to medium in a 4-beat gait.		Dog movement
Trotting	Dog is trotting, defined as a 2-beat gait.		Dog movement
Canter	Dog is cantering, defined as a 3-beat gait.		Dog movement
In flight zone	Dog is within one sheep-body-length of sheep.		Force
Outside of flight zone	Dog more than one sheep-body-length from sheep.		Pressure
Gaze	Dog is looking in fixed direction.	Gaze direction indicates what the dog is most likely to be responding to.	Human–dog interaction
Physical contact	Dog’s head, mouth or sternum makes physical contact with sheep.		Force

Ethogram used for the coding of dog behavior in an in situ pilot study of an Australian abattoir.

**Table A3:** Human Ethogram.

Behavior	Modifiers	Description	Category
Distance from sheep	Outside of flight zone	Handler is further than one sheep body length from any sheep present	Position
Distance from sheep	Inside of flight zone	Handler is within one sheep body length of any sheep present	Position
Position along race/mob	Near head of mob	Handler is within one sheep body length of the sheep at the front of the mob	Position
	Near rear of mob	Handler is within one sheep body length of the sheep at the rear of the mob (sheep furthest from intended direction)	Position
Movement	Same direction as mob	Handler is moving in the same direction as sheep.	Human movement
	Neutral movement	Handler is moving but neither in the same or opposite direction as sheep.	Human movement
	Opposite direction as sheep	Handler is moving in the opposite direction to sheep movement.	Human movement
Arm position	Forearms protracted	Handler has forearms above hipline, upper arms by sides (including arms folded).	Human movement
	Whole arm protracted	Handler has both upper and lower arm raised or outstretched	Human movement
Arm movement	One arm moving	Handler is moving one arm.	Human movement
	Both arms moving	Handler is moving both arms	Human movement
Bag/bell use	Held but not in use	Handler has a bag or bell but is not shaking the object.	Object interaction
	In use	Handler has a bag or bell and is shaking the object.	Object interaction
Gaze direction	Sheep	Time handler spends with face and eyes (if visible) pointing directly at sheep	Object interaction
	Dog	Time handler spends with face and eyes (if visible) pointing directly at dog	Object interaction
Gate manipulation	Handler opens gate—no jamming	Handler opens a gate within or between races, without physically pushing against sheep.	Object interaction
	Handler opens gate—jamming	Handler opens a gate within or between races, gate is physically pushed against one or more sheep.	Object interaction
	Handler shuts gate—no jamming	Handler shuts a gate within or between races without physically pushed against sheep	Object interaction
	Handler shuts gate—jamming	Handler shuts a gate within or between races, gate is physically pushed against one or more sheep.	Object interaction
Foot touch	-	Handler touches sheep with foot.	Animal directed
Touching sheep	-	Handler touches a sheep with one hand.	Animal directed
Grabbing sheep	-	Hander grabs or touches a sheep with both hands.	Animal directed
Touching dog	-	Handler pats, rubs or scratches dog	Animal directed

Ethogram used for the coding of human behavior in an in situ pilot study of an Australian abattoir.
